# Prognostic value of inflammation-based scores in patients receiving radical resection for colorectal cancer

**DOI:** 10.1186/s12885-018-4842-3

**Published:** 2018-11-12

**Authors:** Fang Wang, Wenzhuo He, Chang Jiang, Guifang Guo, Bin Ke, Qiangsheng Dai, Jianting Long, Liangping Xia

**Affiliations:** 1 Sun Yat-sen University Cancer Center; State Key Laboratory of Oncology in South China, 651, Dongfengdong Road, Guangzhou, 510060 China; 2 Department of Oncology, The First Affiliated Hospital of Sun Yat-sen University, 58, the 2nd Zhongshan Road, Guangzhou, 510080 China; 3 Sun Yat-sen University Cancer Center; State Key Laboratory of Oncology in South China; Collaborative Innovation Center for Cancer Medicine, 651, Dongfengdong Road, Guangzhou, 510060 China; 4 Department of Traditional Chinese Medicine, The First Affiliated Hospital of Sun Yat-sen University, 58, the 2nd Zhongshan Road, Guangzhou, 510080 China

**Keywords:** Colorectal cancer, Radical resection, Modified-Glasgow prognostic score, Systemic inflammation score, Albumin-neutrophil-to-lymphocyte ratio, And intratumoural chronic inflammatory cell

## Abstract

**Background:**

The modified Glasgow Prognostic Score (mGPS) and the neutrophil-to-lymphocyte ratio (NLR) are conventional inflammation-based scores for colorectal cancer (CRC). The systemic inflammation score (SIS) has been shown to be more informative than the mGPS in CRC. The albumin-NLR, composed of albumin and the NLR, can also be a candidate for a valuable inflammation score. However, about the utility of the mGPS, SIS, and albumin-NLR for CRC patients who have received radical resections remains unclear.

**Methods:**

This study enrolled 877 CRC patients, who underwent radical surgical resection between January 1, 2007 and December 31, 2014. The prognostic values of the mGPS, SIS, and albumin-NLR were compared by the Kaplan-Meier survival analysis, multivariate Cox regression modelling, and the time-dependent receiver operating characteristic curve analysis (ROC).

**Results:**

In the Kaplan-Meier analysis, all three inflammation scores were significantly associated with overall survival (OS) in the group including all the patients (mGPS, *p* = 0.016; SIS, *p* < 0.001; albumin-NLR, *p* = 0.007) and in the left-sided colon tumour subgroup (mGPS, *p* = 0.029; SIS *p* = 0.0013; albumin-NLR, *p* = 0.001). In the right-sided colon tumour subgroup, only the albumin-NLR was associated with OS (*p* = 0.048). The albumin-NLR was the only independent prognostic factor of the three scores for OS in the multivariate survival analysis.

**Conclusions:**

The albumin-NLR outperformed both the SIS and mGPS in predicting OS in CRC patients undergoing radical resection.

## Background

Colorectal cancer is the leading cause of cancer mortality worldwide [1]. Despite immense efforts in developing advanced treatments for this disease, the overall survival for colorectal cancer remains poor, even in patients who receive resection with curative intent, with only 50% of patients surviving 5 years post-surgery [2].

As commonly recognized, the progression of colorectal tumours is dependent not only on the tumour characteristics alone but also on the systemic environment of the host. Furthermore, there is increasing evidence that both local and systemic inflammatory responses play an important role in the progression of a variety of common solid tumours [3–7].

For systemic inflammation indices, the neutrophil-to-lymphocyte ratio (NLR) and the lymphocyte-to-monocyte ratio (LMR) have been identified as prognostic markers for colorectal cancer [8–10]. Furthermore, the modified Glasgow Prognostic Score (mGPS), which comprises the serum C-reactive protein (CRP) and serum albumin levels, has been demonstrated as a favourable prognostic index for colorectal cancer (CRC) patients [11–13]. Recently, Suzuki et al. developed the systemic inflammation score (SIS), which comprises LMR and serum albumin levels and was found to be superior to the mGPS in predicting prognosis for colorectal cancer patients [14].

For local inflammation factors, tumour-infiltrating lymphocytes (TILs) have been identified as robust factors predicting outcomes in several reports investigating solid tumours [15–18]. In 1986, Jass first proposed TIL as a novel independent prognostic factor for CRC, and this new system was considered superior to the Duke’s staging system [19]. However, the prognostic value of TILs in CRC remains controversial due to a limited number of studies. In addition, the correlation of systemic inflammation indices and local inflammation factors has rarely been studied.

The aim of this study was to compare the prognostic value of the mGPS, SIS, and albumin-NLR, comprising serum albumin and NLR, and to determine the relationship between local and systemic inflammation factors.

## Methods

### Patient selection

A series of 877 CRC patients were enrolled in this study. The patients were treated with radical surgical resection at the Sun Yat-Sen University Cancer Center between January 1, 2007, and December 31, 2014. The inclusion criteria were the following: (1) The patient underwent radical resection at Sun Yat-Sen University Cancer Center for pathologically confirmed American Joint Committee on Cancer (AJCC) stage I to III CRC; (2) the patient received routine analyses of blood, CRP, and serum albumin levels before surgery; and, (3) the resected specimens and pathology slides were stored at our institution. The exclusion criteria were the following: (1) the patient received neoadjuvant chemo-radiotherapy or radiation therapy; (2) the patient experienced acute or chronic inflammation; (3) the patient experienced double cancer; and, (4) the patient’s case had insufficient data.

A routine follow-up was conducted by the follow-up department of Sun Yat-Sen University Cancer Center. Overall survival (OS) was calculated from the time of diagnosis to the date of death or the last follow-up visit.

### Data extraction

The clinical characteristics and serological examination results of all the study patients were collected from medical records. Hematoxylin and eosin (H&E) stained tissue sections from surgically resected tumour specimens were reviewed for all CRC patients. One pathologist evaluated all available sections for each patient and selected the slide with the highest intratumoural chronic inflammatory cell (CIC) density. Two pathologists scored the average CIC density (lymphocytes, neutrophils and plasma cells) in both the central region and the invasive margin of the tumour. Follow-up data were available for all patients, and the latest follow-up was conducted on September 29, 2017. The OS was defined as the time from diagnosis to death from any cause or censored at the date of the last follow-up.

### Inflammation-based incidences

The neutrophil-granulocyte cell count, lymphocyte cell count, monocyte cell count, serum albumin, and CRP levels were recorded before treatment. The mGPS score was established using the serum CPR and albumin levels as previously described [20]. The NLR is equal to the neutrophil count divided by the total lymphocyte count; the LMR is derived from the lymphocyte count divided by the monocyte count. The cut-off points for NLR and LMR were 2.39 and 3.80, respectively, which were derived using the ROC analysis. The SIS, which comprises the LMR and serum albumin, was defined as follows: patients with LMR ≥3.8 and serum albumin ≥39.75 g/L were scored as 0; patients with LMR < 3.8 or serum albumin < 39.75 g/L were scored as 1; and patients with LMR < 3.8 and serum albumin levels < 39.75 g/L were scored as 2. For the albumin-NLR assessment, patients with serum albumin levels ≥39.75 g/L and NLR < 2.39 were assigned a score of 0; patients with either hypoalbuminemia (< 39.75 g/L) or elevated in NLR levels (≥2.39) were scored as 1; and those with both hypoalbuminemia (< 39.75 g/L) and an increase in NLR levels (≥2.39) were scored as 2.

The average CIC density was evaluated in the central region and the invasive margin of the tumour. At the invasive margin of the tumour: a score of 0 indicated no increase in inflammatory cells; a score of 1 denoted a mild and patchy increase of inflammatory cells at the invasive margin but no destruction of the invading cancer cell islets by the inflammatory cells; a score of 2 was given when inflammatory cells formed a band-like infiltration at the invasive margin with some destruction of the cancer cell islets; and a score of 3 denoted a very prominent inflammatory reaction, forming a cup-like zone at the invasive margin with frequent and invariably present destruction of the cancer cell islets. A similar scale was used at the central region, a score of 0 indicated absence of a reaction; a score of 1 indicated a weak reaction; a score of 2 indicated a moderate reaction; and a score of 3 indicated a severe increase in each cell type.

A Crohn’s-like reaction was defined as a transmural lymphoid reaction. It was scored as 0 (absent), 1+ (mild), 2+ (moderate), or 3+ (marked).

All the patients were further divided into either the low-response group or high-response group according to the above inflammation reaction scores. The patients with scores of 0 and 1 were placed in the low-response group, while patients with scores of 2 and 3 were included in the high-response group.

### Statistical analysis

The data were presented as the median values and ranges. The distribution of clinicopathological characteristics according to the different groups was analysed using the Chi-squared or Kruskal-Wallis test, where appropriate. The Kaplan-Meier method and the log-rank test were used to study the impact of different clinical factors on OS. The univariate and multivariate survival analyses had hazard ratios (HRs) calculated using the Cox proportional hazards model. The prognostic ability of the different inflammation scoring methods was compared by generating time-dependent receiver operating characteristic (ROC) curves and by calculating the area under the curve (AUC). The time-dependent ROC curve analysis is used to assess the discriminatory power of continuous markers for time-dependent disease outcomes. It is an extension of the ROC curve and can calculate the AUC and concurrently compare the ROC curves [14]. The sequential AUCs were compared between the mGPS, SIS and albumin-NLR using independent and identically distributed representations [14]. A probability value of *p* < 0.05 was defined as statistically significant. All statistical analyses were performed using the SPSS statistical package version 22.0 (IBM Corp., Armonk, NY, USA) and the R studio version 3.4.2 (R Foundation for Statistical Computing, Vienna, Austria).

## Results

### Patient characteristics

A total of 877 patients were included in the final analysis. The median age of all patients was 59 years (range, 19–88 years). The distribution of sex was 533 (60.8%) male patients and 344 (39.2%) female patients. The baseline clinical characteristics and pathological findings of the patients are summarized in Table 1. There were 89 (10.1%) patients with microsatellite instability (MSI). For the distribution of the TNM stage, 106 (12.1%) patients were stage I, 505 (57.6%) patients were stage II, and 266 (57.6%) patients were stage III. The median OS of patients with CRC was 44.53 months (range: 0.73–123.80 months). The median follow-up period was 46.73 months (range: 0.73–123.80 months).

**Table 1:** Patients’ Clinicopathological Characteristics and Associations with the mGPS, SIS and Albumin-NLR

		mGPS				SIS				Albumin-NLR			
	All Case	0	1	2	P value	0	1	2	*P* value	0	1	2	*P* value
Cases	877	669	123	85		331	331	213		284	385	208	
Age	59(19–88)	59	59	62	0.064	57	60	62	< 0.001	57	60	61	0.001
Sex													
Male	533	391	87	55	0.027	197	194	142	0.122	179	225	129	0.445
Female	344	278	36	30		134	139	71		105	160	79	
Location													
Right-sided colon	326	218	63	45	< 0.001	107	129	90	0.049	92	144	105	< 0.001
Left-sided colon	551	451	60	40		224	204	123		192	241	103	
TNM stage													
I	106	95	6	5	< 0.001	51	38	17	0.065	49	41	16	0.001
II	505	359	89	57		178	191	136		149	216	140	
III	266	215	28	23		102	104	60		86	128	52	
T stage													
1	32	30	0	2	0.021	16	11	5	0.026	10	20	2	< 0.001
2	99	36	8	5		46	35	18		48	32	19	
3	598	446	92	60		219	239	140		181	276	141	
4	148	107	23	18		50	48	50		45	57	46	
N stage													
0	602	447	93	62	0.179	223	226	153	0.739	196	251	155	0.232
1	90	77	7	6		37	36	17		30	43	17	
2	185	145	23	17		71	71	43		58	91	36	
TLN													
< 12	294	242	33	19	0.009	120	115	59	0.105	113	128	53	0.004
> =12	583	427	90	66		211	218	154		171	257	155	
NLN													
< 11	293	237	34	22	0.073	120	112	61	0.184	108	128	57	0.047
> =11	584	432	89	63		211	221	152		176	257	151	
Histology stage													
G1	8	6	2	0	0.08	3	3	2	0.589	1	6	1	0.167
G2	626	489	77	61		244	230	152		208	277	141	
G3	114	89	16	9		44	48	22		36	53	25	
G4	128	85	28	15		40	52	36		39	48	41	
Nerve invasion													
No	725	548	101	76	0.224	261	276	188	0.018	234	308	183	0.049
Yes	152	121	22	9		70	57	25		50	77	25	
Vascular invasion													
No		516	101	68	0.426	253	255	177	0.129	223	287	175	0.026
Yes		153	22	17		78	78	36		61	98	33	
MSI status													
Deficient	89(10.1%)	51	26	12	< 0.001	29	22	38	< 0.001	26	28	35	0.001
Proficient	788(89.9%)	618	97	73		302	311	175		258	357	173	
Neutrophil in central region													
Low	357	304	30	23	< 0.001	167	129	61	< 0.001	147	150	60	< 0.001
High	520	365	93	62		164	204	152		137	235	148	
Neutrophil in invasive margin													
Low	561	445	66	50	0.014	243	205	113	< 0.001	200	251	110	< 0.001
High	316	224	57	35		88	128	100		84	134	98	
Lymphocytes in central region													
Low		264	57	28	0.142	131	138	80	0.661	110	165	74	0.203
High		405	66	57		200	195	133		174	220	134	
Lymphocytes in invasive margin													
Low	427	324	63	40	0.809	162	167	98	0.636	133	208	86	0.01
High	450	345	60	45		169	166	115		151	177	122	
Crohn’s-like													
No	796	624	97	75	< 0.001	311	301	184	0.012	263	355	178	0.013
Yes	78	43	25	10		19	31	28		21	28	29	

*mGPS* modified Glasgow Prognostic Score, *SIS* systemic inflammation score, *albumin-NLR* albumin-neutrophil-to-lymphocyte ratio, *TLN* total lymph nodes resected, *NLN* negative lymph nodes resected, *MSI* microsatellite instability

### Clinicopathological findings

The relationship between patient clinicopathological characteristics and inflammation-based scores is listed in Table 1. The elevated mGPS, SIS and albumin-NLR scores were significantly associated with an advanced T stage (*p* = 0.021, *p* = 0.026, *p* = 0.021), microsatellite instability (MSI) status (*p* < 0.001, *p* < 0.001, *p* = 0.001), and local intratumoural inflammation factors, such as higher neutrophil density in the central region of the tumour (all *p* < 0.001) and the invasive margin of tumour (*p* = 0.014, *p* < 0.001, *p* = 0.01), as well as a Crohn’s-like reaction (*p* < 0.001, *p* < 0.001, *p* = 0.013). The elevated mGPS score was significantly associated with the male sex (*p* = 0.024), left-sided colon lesions (*p* < 0.001), an advanced disease stage (*p* < 0.001), and a lower number of lymph nodes resected (*p* = 0.009). The increased SIS and albumin-NLR scores correlated with older age (both *p* < 0.001), left-sided colon lesions (*p* = 0.049, *p* < 0.001), and a higher degree of lymphatic invasion (*p* = 0.018, *p* = 0.049). An increased albumin-NLR score was associated with an advanced disease stage (*p* = 0.001), a higher number of total and negative lymph nodes resected (*p* = 0.004 and *p* = 0.047, respectively), a higher degree of vascular invasion (*p* = 0.026), and a higher density of lymphocytes in the tumour invasive margin (*p* = 0.01).

### Kaplan-Meier analysis

**Fig. 1:**
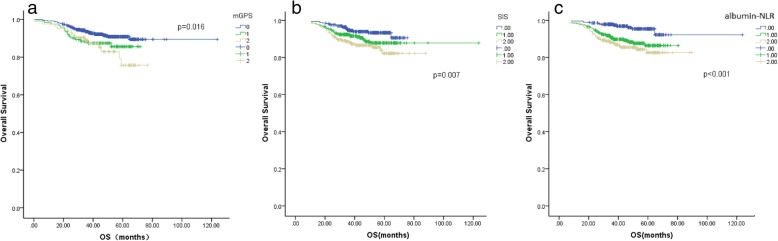
Kaplan-Meier and multivariate analysis of mGPS, SIS and albumin-NLR in the group including all CRC patients. The mGPS, SIS and albumin-NLR were significantly associated with OS (mGPS, *p* = 0.016; SIS, *p* < 0.001; albumin-NLR, *p* = 0.007). The lines of albumin-NLR and SIS separated very well (**b**, **c**), but overlapped each other according to mGPS (**a**)

**Fig. 2:**
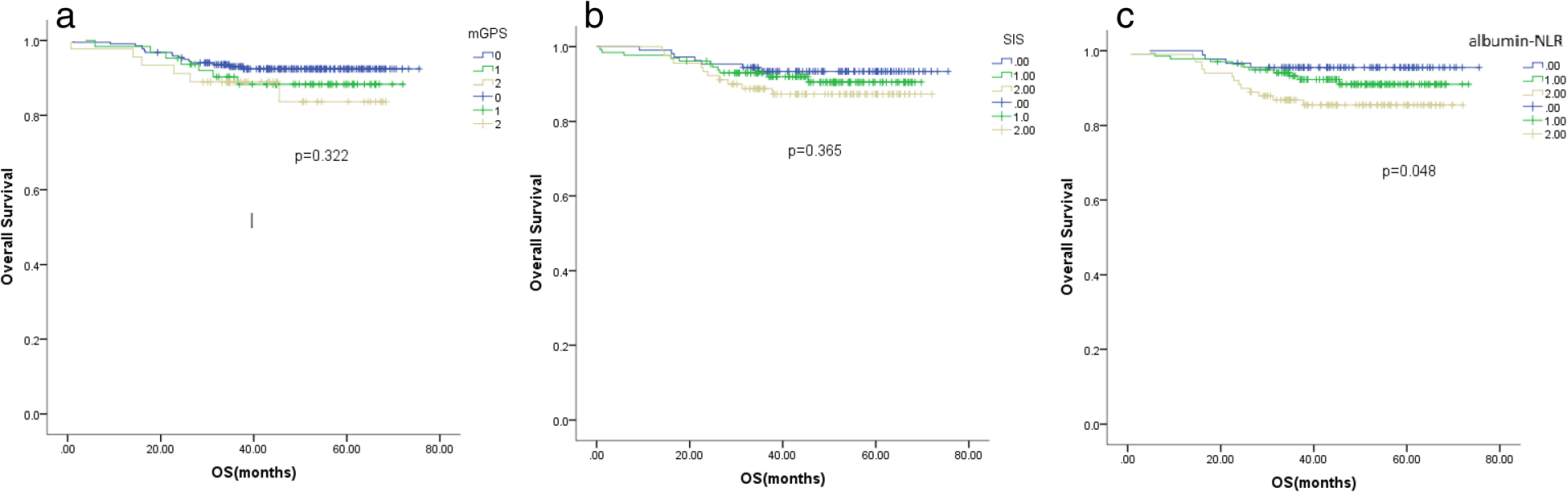
Kaplan-Meier analysis of the mGPS, SIS and albumin-NLR in the right-sided colon cancer patients group. Only the albumin-NLR was significantly associated with OS (*p* = 0.048), with the survival curves being well separated (**c**), the lines of mGPS and SIS crossed each other (**a**, **b**)

**Fig. 3:**
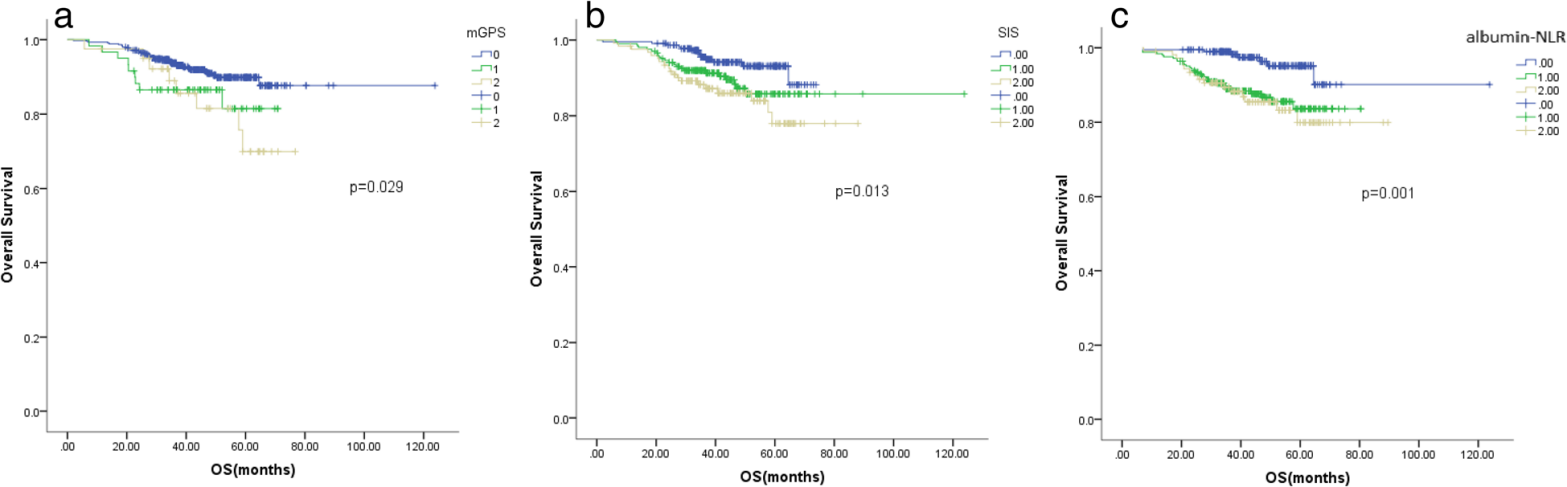
Kaplan-Meier analysis of the mGPS, SIS and albumin-NLR in the group of patients with left-sided colon cancer. All three score types were independent factors for OS (mGPS, *p* = 0.029; SIS *p* = 0.0013; albumin-NLR, *p* = 0.001). The lines scored 0 seperated with the lines scored 1 and 2 in mGPS, SIS, albumin-NLR; but, the lines scored 1 overlaped the lines scored 2 in mGPS, SIS and albumin-NLR (**a**, **b**, **c**)

The Kaplan-Meier curves for the OS rate were divided into three groups according to the mGPS, SIS, and albumin-NLR scores. The influence of the above three factors on the OS of the study patients is shown in Fig. 1. All three inflammation score types were significantly associated with OS (mGPS, *p* = 0.016; SIS, *p* < 0.001; albumin-NLR, *p* = 0.007). It should be noted that the curves of the mGPS overlapped. In the right-sided colon tumour subgroup, only the albumin-NLR was associated with OS (*p* = 0.048), with the survival curves being well separated (Fig. 2). In the left-sided colon tumour subgroup, all three score types were independent factors for OS (mGPS, *p* = 0.029; SIS *p* = 0.0013; albumin-NLR, *p* = 0.001) (Fig. 3).

**Fig. 4:**
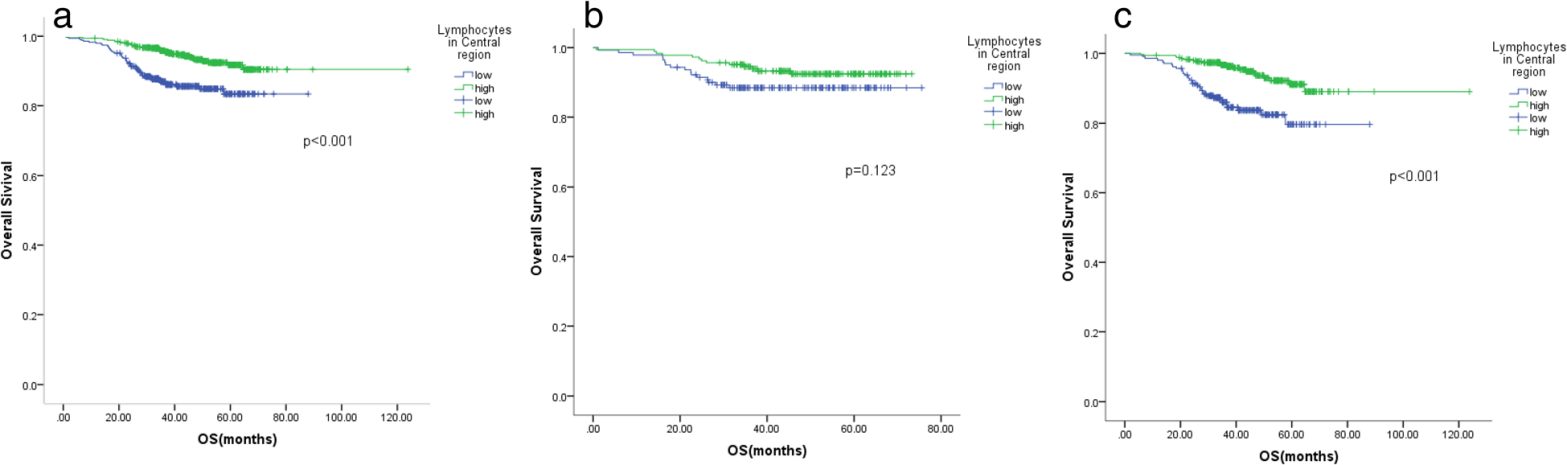
Kaplan-Meier analysis of the lymphocytes in the central region in the group including all CRC patients and the right-sided and left-sided colon tumour subgroups. The lymphocytes in the central region of the tumour were significantly associated with OS in the group including all patients and the left-sided colon cancer group (a, c). **a**: the group including all patients, **b**: the group of patients with right-sided colon cancer, **c**: the group of patients with left-sided colon cancer

**Fig. 5:**
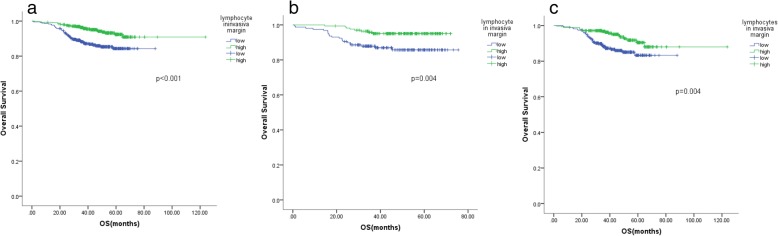
Kaplan-Meier analysis of the lymphocytes in the invasive margin the group including all CRC patients and the right-sided and left-sided tumour subgroups. The lymphocytes in the tumour invasive margin were significant factors for OS in all three groups (a, b, c). **a**: the group including all patients, **b**: the group of patients with right-sided colon cancer, **c**: the group of patients with left-sided colon cancer

For the local tumour-infiltrating inflammation measure, lymphocytes in the central region of the tumour were significantly associated with OS in the group including all patients and the left-sided colon cancer group (Fig. 4), while lymphocytes in the tumour invasive margin were significant factors for OS in all three groups (Fig. 5). Neutrophils in the central region of the tumour were significant factors for OS in the right-sided colon cancer subgroup (*p* = 0.042). The neutrophil count in the central region and the invasive margin of the tumour, as well as a Crohn’s-like reaction, failed to predict OS in the other patient subgroups.

### Univariate and multivariate survival analyses

For the univariate survival analysis, factors such as the mGPS, SIS, and albumin-NLR, age, TNM stage, T stage, N stage, total number of lymph nodes resected (TLN), number of negative lymph nodes resected (NLN), lymphatic invasion, vascular invasion, lymphocytes in the tumour invasive margin, and lymphocytes in the central region of the tumour, were all identified as significant prognostic factors for OS. All these factors with *p* < 0.05 were included in the multivariate survival analysis. The albumin-NLR, age, N stage, and the total lymph nodes resected (TLN) were demonstrated to be independent prognostic factors for OS (Table 2).

**Table 2:** Univariate and Multivariate Analysis of Clinicopathological Characteristics in Relation to the Overall Survival in Patients with CRC Undergoing Curative Resection

	Univariate		Multivariate	
	HR(95% CI)	*p*	HR(95% CI)	*p*
mGPS	1.49(1.129–1.965)	0.005	1.223(0.867–1.725)	0.253
SIS	1.5441.173–2.033)	0.002	0.869(0.562–1.345)	0.529
Albumin-NLR	1.812(1.35–2.433)	< 0.001	2.112(1.314–3.395)	0.002
Age	1.025(1.00701.044)	0.007	1.032(1.012–1.052)	0.001
TNM stage	2.251(1.553–3.263)	< 0.001	0.845(0.419–1.705)	0.638
T stage	1.674(1.162–2.412)	0.006	1.332(0.877–2.023)	0.179
N stage	1.759(1.397–2.241)	< 0.001	1.760(1.142–2.712)	0.01
TLN	0.425(0.276–0.654)	< 0.001	0.375(0.164–0.857)	0.02
NLN	0.383(0.248–0.591)	< 0.001	1.050(0.460–2.397)	0.908
Vascular invasion	2.501(1.606–3.893)	< 0.001	0.557(0.903–2.686)	0.111
Peripheral nerve invasion	1.732(1.046–2.868)	0.033	1.169(0.674–2.025)	0.578
Lymphocytes in invasive margin	0.396(0.249–0.63)	< 0.001	0.694(0.385–1.249)	0.223
Lymphocytes in central region	0.405(0.261–0.627	< 0.001	0.680(0.394–1.174)	0.167

*mGPS* modified Glasgow Prognostic Score, *SIS* systemic inflammation score, *albumin-NLR* albumin- neutrophil-to-lymphocyte ratio; *TLN* total lymph nodes resected, *NLN* negative lymph nodes resected, *HR* hazard ratio, *CI* confidence interval

### Time-dependent ROC curve analysis

**Fig. 6:**
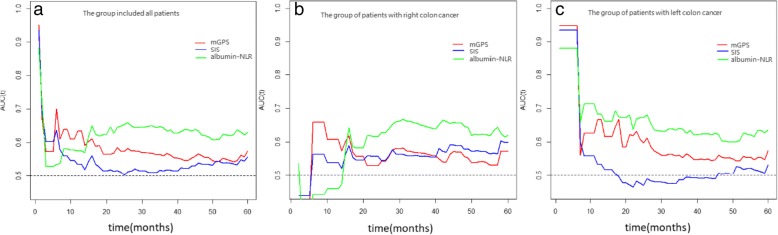
Time-dependent ROC curves for the mGPS, SIS and albumin-NLR. The horizontal axis represents the year after surgery and the vertical axis represents the estimated area under the ROC curve for survival at the time of interest. Red, blue and green solid lines represent the estimated AUC of the mGPS, SIS and albumin-NLR, respectively. The albumin-NLR was superior to the mGPS and SIS in predicting the 5-year survival rate of CRC patients in all three groups. **a**: the group including all patients, **b**: the group of patients with right-sided colon cancer, **c**: the group of patients with left-sided colon cancer

The time-dependent ROC curve was used to compare the sequential trends of the albumin-NLR, SIS, and mGPS scores, according to the hazard ratios for OS. The time-dependent ROC curve was the integration of the estimated AUC at each time point. In the group including all CRC patients, the time-dependent ROC curve of the albumin-NLR crossed the curves of both the SIS and mGPS at the 15th-month point after surgery and was continuously superior to the other two curves in predicting the 5-year survival rate of patients. Moreover, the ROC curve of the mGPS was superior to the SIS curve. In the right-sided colon cancer subgroup, the albumin-NLR was the most robust inflammation factor for the sequential prediction of OS; at the same time, the time-dependent ROC curve of the mGPS was superior to that of the SIS. For the left-sided colon cancer subgroup, the time-dependent ROC curve of the albumin-NLR was also superior to that of the SIS and mGPS (Fig. 6).

## Discussion

Our research was the first study to compare the SIS, mGPS and albumin-NLR as prognostic indicators for OS in CRC patients with radical resection. We demonstrated that the albumin-NLR was a more powerful prognostic factor for OS than the other two. In addition, we also analysed the relationship between inflammation-based scores and local inflammation indices, which was an important supplement to inflammation-based prognostic indices.

With the emergence of immunotherapy, the immune system status and inflammation severity have become the focus of many studies. The traditional TNM stage is limited in this regard, indicating the need for a more robust prognostic system. Inflammation factors can quantify and characterize the inflammatory state of the patient and the general immune system status.

The NLR has been thoroughly studied and identified as an independent prognostic factor for OS in multiple solid tumour sites [21], including CRC [8]. Several studies have demonstrated that the NLR was the only independent prognostic factor for OS in CRC patients among the LMR, platelet-to-lymphocyte ratio (PLR) and the prognostic nutritional index (PNI) [9, 20]. However, the LMR has been shown to be a valuable prognostic factor for OS in several solid tumour types [22–24]. Previously, Chan et al. demonstrated that the LMR was superior to both the NLR and PLR as a predictor of OS in resectable CRC patients [10]. The mGPS criterion is a well-known inflammation index for OS in CRC [13, 25]. However, Suzuki et al. recently discovered the SIS to be superior to the mGPS in CRC patients who received curative surgical resection [14]. At present, there is no consensus as to which inflammatory biomarker is the most clinically useful or the best predictor of prognosis for CRC.

The mGPS and SIS are composed of a CRP and a simple inflammation factor. As the NLR is a well-known inflammation factor for CRC patients and is also compared to the LMR, we constructed the albumin-NLR by incorporating CRP and the NLR.

In our study, the albumin-NLR showed the optimal Kaplan-Meier curves in the group including all CRC patients, and it was also the only significant marker for OS in the right-sided colon cancer subgroup. In the left-sided CRC subgroup, the three inflammation factors were almost equally robust in the Kaplan-Meier analysis. The albumin-NLR proved to be the only independent prognostic index for OS by the multivariate Cox proportional-hazard regression analysis. Furthermore, the time-dependent ROC curve showed that the albumin-NLR was continually superior to the mGPS and SIS in the three patient groups in our study. The AUC of the albumin-NLR for OS ranged from 0.5252 to 0.877 and ranged from 0.602 to 0.803 in the group including all patients and the left-sided colon group, respectively. In the right-sided colon group, the time-dependent ROC curve of the albumin-NLR was superior to the mGPS and SIS starting at the 16th month after surgery, and the AUC of the albumin-NLR for OS ranged from 0.579 to 0.669 since then. Although the AUC of the albumin-NLR in our study was less than 0.7, it was in accordance with the AUC of the classic prognostic factors reported for CRC patients, such as CEA [26, 27].

The indices derived from the comprehensive blood tests are a reflection of the inflammation status generated both at the local level and systemically before resection. It has been reported that tumour-infiltrating lymphocytes and neutrophils correlated with peripheral blood lymphocytes and neutrophils [17]. The local tumour-infiltrating inflammatory cells correlated to the patient outcomes in our study. The patients with high neutrophil density in the central region of the tumour and the tumour invasive margin correlated with higher albumin-NLR and SIS score groups. Patients with lower lymphocyte counts in the tumour invasive margin showed a higher albumin-NLR score, but the other two inflammatory markers showed no relationship. Patients without local Crohn’s-like reactions had higher albumin-NLR and SIS scores. There have been previous reports of high neutrophil and lymphocyte counts in the central region of the tumour and the tumour invasive margin being related to longer survival [28]. Our findings also showed that patients with higher lymphocyte counts in the central region of the tumour and the tumour invasive margin were related to better patient survival. The albumin-NLR was the only inflammation score type found to predict OS, indicating that this measure may better reflect the status of the local tumour-infiltrating inflammatory cells.

The patients with MSI had higher albumin-NLR and SIS scores. CRC patients with MSI are known to be more sensitive to immunotherapy [29]. The MSI status was associated with a high mutational burden and immune infiltration [29], which provided recognizable cancer antigens for the immune system. The active immune system can be reflected by the local and systemic inflammation levels during malignancy. Moreover, inflammation indices that are derived from the peripheral blood better reflect the current immune status than other markers, such as CRP.

C-reactive protein is an acute temporal response protein, which reflects acute trauma or the inflammatory condition of the patient, instead of the local or systemic inflammation status when cancer is present. The markers derived from the peripheral blood were more effective at this point. We also analysed the prognostic value of the NLR and LMR for OS by the Kaplan-Meier method. The NLR was a significant factor for OS (*p* = 0.023), but the LMR failed to predict OS (*p* = 0.065). This result lends some explanation for why the albumin-NLR was superior to the SIS in terms of OS in our study.

The accumulated data suggest that inflammatory markers are associated with pathological features and prognosis in CRC patients. The pathological factors, such as the T stage, N stage, TLN, and NLN are well-known prognostic factors. For our study, we enrolled only limited stage patients, thereby requiring the analysis of the prognostic power of the three inflammation scoring methods in subgroups of the T stage, N stage, TLN, and NLN, by the Kaplan-Meier method. The albumin-NLR curve for OS was the most robust. From the results of the Kaplan-Meier analysis, it can be concluded that the albumin-NLR was a powerful prognostic factor for OS. The albumin-NLR can further predict the prognosis for the T stage, N stage, TLN, and NLN subgroups and should be considered as a supplement to TNM staging.

There were several limitations to this study. First, this report had a retrospective study design, which may induce some selection bias. Second, most of the study patients were administered routine adjuvant chemotherapy, but since the chemotherapy data were incomplete, we could not thoroughly analyse any possible relationship between treatment agents and inflammation factors. Moreover, the data regarding local macrophages was not available. Finally, since the albumin-NLR is a novel inflammation score, more research is needed to validate this factor’s prognostic value and further applications.

## Conclusions

In conclusion, the albumin-NLR inflammation scoring method outperformed both the SIS and mGPS in predicting survival in CRC patients undergoing resection, indicating that albumin-NLR is a useful inflammatory marker. Further prospective studies should be conducted to confirm these results.

## Abbreviations

AJCC: American Joint Committee on Cancer
albumin-NLR: Albumin-neutrophil-to-lymphocyte ratio
CIC: Intratumoural chronic inflammatory cell
CRC: Colorectal cancer
CRP: C-reactive protein
HRs: Hazard ratios
LMR: Lymphocyte-to-monocyte ratio
mGPS: Modified Glasgow Prognostic Score
MSI: Microsatellite instability
NLN: Negative lymph nodes resected
PLR: Platelet-to-lymphocyte ratio
PNI: Prognostic nutritional index
ROC: Receiver operating characteristics analysis
SIS: Systemic inflammation score
TILs: Tumour-infiltrating lymphocytes
TLN: Total lymph nodes resected
